# Altered Behavioral and Autonomic Pain Responses in Alzheimer’s Disease Are Associated with Dysfunctional Affective, Self-Reflective and Salience Network Resting-State Connectivity

**DOI:** 10.3389/fnagi.2017.00297

**Published:** 2017-09-14

**Authors:** Paul A. Beach, Jonathan T. Huck, David C. Zhu, Andrea C. Bozoki

**Affiliations:** ^1^D.O., Ph.D. Training Program, Michigan State University College of Osteopathic Medicine East Lansing, MI, United States; ^2^Neuroscience Program, Michigan State University East Lansing, MI, United States; ^3^Department of Radiology, Michigan State University East Lansing, MI, United States; ^4^Department of Psychology, Michigan State University East Lansing, MI, United States; ^5^Department of Neurology & Ophthalmology, Michigan State University East Lansing, MI, United States

**Keywords:** Alzheimer’s disease, pain, resting-state fMRI, PAINAD, autonomic response

## Abstract

While pain behaviors are increased in Alzheimer’s disease (AD) patients compared to healthy seniors (HS) across multiple disease stages, autonomic responses are reduced with advancing AD. To better understand the neural mechanisms underlying these phenomena, we undertook a controlled cross-sectional study examining behavioral (Pain Assessment in Advanced Dementia, PAINAD scores) and autonomic (heart rate, HR) pain responses in 24 HS and 20 AD subjects using acute pressure stimuli. Resting-state fMRI was utilized to investigate how group connectivity differences were related to altered pain responses. Pain behaviors (slope of PAINAD score change and mean PAINAD score) were increased in patients vs. controls. Autonomic measures (HR change intercept and mean HR change) were reduced in severe vs. mildly affected AD patients. Group functional connectivity differences associated with greater pain behavior reactivity in patients included: connectivity within a temporal limbic network (TLN) and between the TLN and ventromedial prefrontal cortex (vmPFC); between default mode network (DMN) subcomponents; between the DMN and ventral salience network (vSN). Reduced HR responses within the AD group were associated with connectivity changes within the DMN and vSN—specifically the precuneus and vmPFC. Discriminant classification indicated HR-related connectivity within the vSN to the vmPFC best distinguished AD severity. Thus, altered behavioral and autonomic pain responses in AD reflects dysfunction of networks and structures subserving affective, self-reflective, salience and autonomic regulation.

## Introduction

Several experimental studies probing behavioral indicators of pain in Alzheimer’s disease (AD) patients have found evidence of increased responsiveness, relative to healthy seniors (HS; Porter et al., 1996; Cole et al., 2006; Kunz et al., 2007, 2009; Jensen-Dahm et al., 2014; Beach et al., 2015, 2016). On the other hand, autonomic pain responses become diminished, particularly as AD worsens (Porter et al., 1996; Rainero et al., 2000; Benedetti et al., 2004; Beach et al., 2015), suggesting reduced pain processing and/or central autonomic dysfunction in AD (Rainero et al., 2000; Plooij et al., 2011). Understanding the neural mechanisms of AD’s differential effects on various components of pain is challenging, but necessary in order to improve patient pain assessment and treatment. Our understanding of pain in AD would thus benefit from examining the pain-autonomic relationship more closely.

Pain-autonomic interactions are widespread, particularly supraspinally. Here, viscero-sensory and central autonomic network (CAN) structures overlap and interconnect extensively with medial pain structures, which mediate pain affect, behavioral motivation and cognition (Price, 2000, 2002). This integration of physiologic, affective-behavioral and cognitive processes thus allows for cohesive adaptive responses to noxious stimuli. Key anatomic sites of pain-autonomic interaction include brainstem (e.g., rostral ventral medulla, solitary tract, parabrachial nuclei, periaqueducal gray), hypothalamic, subcortical (amygdala) and cortical (insula, cingulate and ventromedial prefrontal) structures (Benarroch, 2006). In healthy individuals acute pain ratings and pain behaviors are thus generally well-correlated with sympathetic responses like increased heart rate (HR) and skin conductance (Dowling, 1983; Puntillo et al., 1997; Turpin et al., 1999; Loggia et al., 2011; Kyle and McNeil, 2014). The importance of pain-autonomic integration is exemplified by their alteration in chronic pain conditions (e.g., fibromyalgia and headache disorders) as well as in autonomic disorders (e.g., pure autonomic failure and multiple system atrophy; Bleasdale-Barr and Mathias, 1998; Mathias et al., 1999; Solano et al., 2009; Koenig et al., 2015).

Imaging and pathological studies confirm that many cortical (e.g., ventromedial prefrontal, insular) and subcortical structures (parabrachial, periaqueductal gray) subserving pain processing and autonomic regulation are targeted by AD (Chu et al., 1997; Parvizi et al., 2000; Rüb et al., 2001; Scherder et al., 2003). However, the effects of AD pathology on pain processing and autonomic function are not immediately apparent. AD patients experiencing acute experimental pain have similar EEG responses (Gibson et al., 2001; Jensen-Dahm et al., 2016) as well as greater fMRI activation and temporal synchronicity (i.e., functional connectivity) of prefrontal and medial pain structures compared to HS (Cole et al., 2006, 2011). It may be that dysfunctional pain-memory and top-down prefrontal inhibitory processing is responsible for apparently increased pain sensitivity in AD patients (Cole et al., 2006, 2011; Oosterman et al., 2009, 2014; Kunz et al., 2015). What of autonomic function, then? Autonomic dysfunction in AD patients is described by numerous studies (Vitiello et al., 1993; Burke et al., 1994; Algotsson et al., 1995; Giubilei et al., 1998; Idiaquez et al., 2002; Zulli et al., 2005; de Vilhena Toledo and Junqueira, 2008; Idiaquez and Roman, 2011; Zakrzewska-Pniewska et al., 2012; Struhal et al., 2014; Jensen-Dahm et al., 2015; Kim et al., 2015); many studies found evidence of severity-dependent (Idiaquez et al., 2002; Zulli et al., 2005), centrally mediated sympathetic dysfunction during various autonomic maneuvers (Burke et al., 1994; Zakrzewska-Pniewska et al., 2012; Struhal et al., 2014; Jensen-Dahm et al., 2015). These general autonomic findings are antiparallel to those of altered pain responses in AD patients, suggesting central autonomic dysfunction leads to the disconnection between behavioral and autonomic pain responses in patients. However, no studies have attempted to test this notion in the context of AD patient brain function.

Recent work using resting-state fMRI (rs-fMRI) has shown that baseline functional connectivity between somatosensory, prefrontal and medial pain structures strongly influences somatic sensation, pain perception and even autonomic function (Boly et al., 2007; Ziegler et al., 2009; Ploner et al., 2010; Fan et al., 2012; Haag et al., 2015). Resting connectivity strength measures also correspond well with task-based fMRI activation in both AD and HS populations (Zamboni et al., 2013), further indicating an influence of resting-state network (RSN) connectivity on pain processing. Key pain processing and CAN structures are considered core hubs of three RSNs in particular: the default mode network (DMN) with the posterior cingulate, precuneus, and retrosplenial cortex; the salience network (SN) with the middle cingulate, ventromedial prefrontal cortex (vmPFC) and insula; and temporal limbic network (TLN) including temporal pole and amygdala. Altered connectivity within and between the DMN, SN and TLN is implicated in chronic pain disorders (Baliki et al., 2008; Napadow et al., 2010; Farmer et al., 2012; Loggia et al., 2013; Yao et al., 2013; Zamboni et al., 2013) as well as AD (Greicius et al., 2004; Seeley et al., 2009; Brier et al., 2012). These observations suggest abnormal resting connectivity within or between pain/autonomic-related RSNs and their associated structures may, in part, facilitate altered pain responses in AD.

The primary aim of this study was to further elucidate the neural underpinnings of altered pain responses in AD patients using rs-fMRI. To do so, we scanned a subset of AD and HS subjects who participated in a prior study of behavioral and autonomic acute pressure pain responses (Beach et al., 2015). This allowed us to evaluate the relationship between RSN connectivity and pain behavior differences between AD patients and HS. We also investigated how RSN connectivity was associated with autonomic response changes across a spectrum of AD severity. These analyses emphasized examining connectivity of RSNs implicated first on pain processing and second the CAN. In so doing we investigated both voxelwise (RSN to whole brain) and between network connectivity measures.

## Materials and Methods

### Subjects

**Figure 1:**
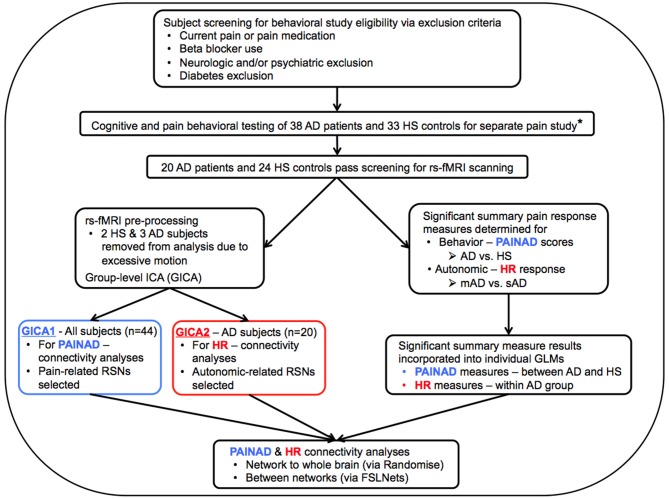
Flow chart describing general study methods. AD, Alzheimer’s disease; HS, Healthy senior; rs-fMRI, resting-state functional magnetic resonance imaging; PAINAD, Pain Assessment in Advanced Dementia scale; HR, Heart Rate; mAD, mild AD; sAD, severe AD; RSNs, Resting-State Networks; GLM, General Linear Model; *Beach et al. (2015).

Twenty-three patients with diagnosed probable AD (14 ♀) and 26 HS subjects (16 ♀), all of whom took part in a larger behavioral study of pain responses in AD (Beach et al., 2015), participated in this study. However, three AD and two HS subjects were not utilized in imaging analyses due to excessive movement during scanning, leaving 20 AD and 24 HS. General subject demographics reflecting subjects utilized in the current study are found in Table 1; general study methods are further found in Figure 1. HS subjects were recruited through senior newsletters and local AD support groups. HS were included only if they had no current pain or history of subjective memory complaint. AD subjects were recruited through the outpatient Cognitive and Geriatric Neurology clinic at Michigan State University. Diagnosis of probable AD was made by a geriatric neurologist (ACB) based on DSM-IV (American Psychiatric Association, 2013) and NINCDS-ADRDA (McKhann et al., 1984) criteria. General study exclusion included history of: Type II diabetes, history of stroke or transient ischemic attack, central or peripheral neuropathy and diagnosis of neurological (e.g., seizure disorder) or psychiatric disorders (e.g., major depression, schizophrenia) other than AD. All participants were screened for baseline pain via subject interview, chart review, or caregiver discussion as inclusion required abstinence from analgesics for 24 h prior to study. We excluded individuals with current arthritic pain, those with a history of arthritis in the distal forearms (the stimulus application region), and those taking daily arthritic pain medication. As our index of autonomic response was HR, individuals taking beta-adrenergic and AV-nodal calcium channel blocking medications were also excluded. Though effects of acetylcholinesterase inhibitors (AChEI) and selective serotonin reuptake inhibitor (SSRI) anti-depressants on autonomic function have been documented (Siepmann et al., 2003; Masuda, 2004; Licht et al., 2010; da Costa Dias et al., 2013), their use was not exclusionary. Recent work suggests AChEI effects are limited to initial administration (Isik et al., 2010; Umegaki and Khookhor, 2013) and likely unrelated to general autonomic dysfunction in AD (Kim et al., 2015; Nonogaki et al., 2017). SSRI effects were also previously found to be non-contributory to autonomic dysfunction in AD (Jensen-Dahm et al., 2015).

**Table 1:** Subject demographics (mean ± standard deviation).

	HS (*n* = 24)	AD (*n* = 20)	*p*^ψ^	mAD (*n* = 13)	sAD (*n* = 7)	*p*^δ^
Age (years)	75.1 (6.7)	76.5 (8.6)	0.54	78.1 (5.7)	73.6 (12.3)	0.27
Gender (F | M)	16 | 8	14 | 6	0.54	9 | 4	5 | 2	0.92
MMSE	29.1 (1.0)	15.3 (7.6)	**<0.001**	20.2 (3.5)	6.3 (3.7)	**<0.001**
SIB-S	- - -	41.6 (9.4)	- - -	47.1 (3.5)	31.3 (8.3)	** <0.001**
CSDD	1.21 (1.3)	8.6 (3.8)	**<0.001**	8.1 (4.0)	9.6 (1.3)	0.39
FAQ	- - -	18.5 (8.4)	- - -	14.1 (7.2)	26.1 (3.0)	**0.001**
Baseline HR (bpm)	69.9 (8.4)	68.2 (11.4)	0.56	69.3 (11.9)	66.2 (11.1)	0.68
AChEI (%)	- - -	80.0	- - -	92.3	57.1	0.06
SSRI (%)	12.3	60.0	**0.003**	53.8	71.4	0.44

*HS, healthy senior controls; AD, Alzheimer’s disease; mAD, mild/moderate Alzheimer’s disease (MMSE 23–11); sAD, severe Alzheimer’s disease (MMSE = 10); MMSE, Mini Mental State Examination; CSDD, Cornell Scale for Depression in Dementia (normal range 0–12); FAQ, Functional Activities Questionnaire; HR, heart rate; SIB-S, Severe Impairment Battery-Short Form; AChEI, acetylcholine esterase inhibitor; SSRI, selective serotonin reuptake inhibitor. Between-group testing of age, MMSE, Cornell, and baseline heart rate via univariate ANOVA; gender, AChEI, and SSRI testing via Chi-square. ψ Indicates between HS and AD ANOVA testing. δ Indicates between mAD and sAD subgroup ANOVA testing. Bold indicates significant group difference at *p* < 0.01. Note: sample sizes reflect those subjects not removed from analyses due to excessive motion*.

Once subjects passed screening they underwent neuropsychological testing, including completion of Mini-Mental State Examination (MMSE; Folstein et al., 1975), and Cornell Scale for Depression in Dementia (CSDD; Alexopoulos et al., 1988). The MMSE is subject to floor effects with increasing severity of dementia. As such, the short form of the Severe Impairment Battery (SIB-S; Saxton et al., 2005) was utilized to better understand cognitive heterogeneity within the AD patient group. AD subjects were also tested for their instrumental activities of daily living (IADLs) through the Functional Activities Questionnaire (FAQ; Pfeffer et al., 1982), a proxy measure in which increasing scores describe worsening IADL ability (max. 30). Mean demographics and scores for neuropsychological tests are found in Table 1. No subjects had a CSDD score indicative of probable depression (>12; Alexopoulos et al., 1988). Of note: as in a prior study of the authors’ examining severity dependent effects of pain responses in AD (Beach et al., 2015), we defined mild/moderate AD (mAD) as MMSE 11–23 and severe AD (sAD) as MMSE ≤10.

Testing procedures were conducted in accordance with the Declaration of Helsinki and were approved by the Michigan State University Internal Review Board. Written informed consent was obtained for all HS as well as AD subjects via named guardians or health care proxies identified as a power of attorney for health care. We obtained assent from all participants before behavioral testing and MRI scanning.

### Procedures

#### Behavioral Testing

The study took place over two sessions. First, subjects underwent behavioral testing as part of an expanded examination of pain responses in AD (Beach et al., 2015, 2016). Mechanical pressure was applied to the volar surface of the distal forearm (2–5 cm from the wrist) using a Force Dial FDK 20 Force Gauge (Wagner Instruments, Greenwich, CT, USA), which allows accurate recording of pressure (kg/cm^2^; see Supplementary Figure S1). The device, scaled in units of “kg,” is fitted with a 1 cm wide rubber disc to prevent skin abrasion. Subjects were seated, upright, during testing. Stimuli ranged from 1 kg to 5 kg in intensity. Each intensity was repeated four times, between the right and left forearms, in a pseudorandom fashion with the order determined once for use in all subjects. Stimulus order was limited by the following rules: no intensity could occur more than twice, sequentially; any sequential intensity repetition could not occur on the same arm. Stimuli were applied at a rate of ~1 kg/s to peak intensity. Pressures were held at peak intensity for 5 s prior to an ~50 s interstimulus interval. A single investigator (PAB) performed all pressure testing. Continuous video recordings during testing allowed for coding of autonomic responses and pain behaviors. Stimulus onset and offset was marked audibly.

As in a prior behavioral study by the authors (Beach et al., 2015), acute pain behaviors were scored during the 5 s stimulus period via the Pain Assessment in Advanced Dementia (PAINAD) scale, an observational pain scale validated for assessing pain in long-term and acute care settings (Warden et al., 2003; Hutchison et al., 2006; Zwakhalen et al., 2006; DeWaters et al., 2008; Herr et al., 2010; Herr, 2011; Guo et al., 2015). The full PAINAD measures five behavioral domains: breathing, consolability, negative vocalizations, facial expressions and bodily responses. Each PAINAD domain score ranges from zero to two for a maximum combined score of 10. Because breathing and consolability are considered poor indicators of pain (van Iersel et al., 2006; Zwakhalen et al., 2006; Schuler et al., 2007; Herr et al., 2008) these domains were not included the authors’ prior behavioral studies. However, for the current study pain behavioral responses were re-scored using the full PAINAD to prevent issues with instrument validity. The use of the PAINAD, vs. more experimental methods such as the Facial Action Coding System (FACS; Ekman et al., 2002), was based on its clinical utility and strong correlation with both FACS measures and subjective pain ratings (Beach et al., 2016). Its use here thus allowed for an improved understanding of how changes in clinically relevant and measurable pain behaviors are related to altered RSN connectivity. PAINAD rater training took place via an online resource whereby trainees viewed and scored videos of cognitively intact and impaired elderly individuals prior to feedback and score explanation (Horgas and Miller, 2008). A single trained rater (JTH), blinded to stimulus order and group designation, scored video recorded sessions. PAINAD intra-rater reliability was strong (intraclass correlation coefficient 0.86) and subject internal consistency was high (Crohnbach’s alpha 0.84). The PAINAD also correlated strongly (*r* = 0.56) with subjective pain report of mAD subjects (Beach et al., 2016), suggesting maintenance of the tool’s construct validity.

A portable infrared monitor (ePulse2™—Impact Sports Technologies), attached just above the elbow, displayed HR throughout testing. Responses were video recorded and reviewed later for scoring. HR was measured on a fixed time window of every 5 s. A given response was determined by subtracting the HR at stimulus onset (baseline) from the maximum response within 30 s after offset, resulting in a positive or negative response. Return to resting HR occurred during the interstimulus interval.

#### fMRI Scanning

In a separate session up to 1 week later, anatomical and rs-fMRI data were collected with a GE 3.0 Tesla Signa HDx MR scanner (GE Healthcare, Waukesha, WI, USA) with an 8-channel head coil. Anatomical scanning involved collection of 180 T1 weighted sagittal volumetric images (TE = 3.8 ms, TR = 8.6 ms, time of inversion = 831 ms, flip angle = 8°, field of view = 25.6 cm × 25.6 cm, matrix size = 256 × 256, slice thickness = 1 mm, receiver bandwidth = ±20.8 kHz). Next, subjects underwent two 7-min resting-state functional echo planar image scans under dimly lit conditions (TE = 27.7 ms. TR = 2500 ms, flip angle = 80°, field of view = 220 mm, matrix 64 × 64 voxels, 168 brain volumes via 36 contiguous axial 3 mm axial slices). Scanning sessions, including anatomical and resting-state scans, was 30 min. One AD subject was unable to complete the second resting-state scan. Prior to each scan subjects were instructed to hold still as much as possible, with their eyes open and stay awake. Wakefulness was monitored live during resting-state scanning through an MR compatible eye camera, which was attached to the head coil.

### Sample Size

The current study utilized a subset of patients that took part of a larger examination of pain response differences (including PAINAD and HR measures) among HS and those with AD of varying severity. An *a priori* power analysis for the aforementioned parent study indicated that 15 subjects per group (HS, mAD and sAD) would be sufficient to reach at least a small effect (*d* = 0.3) at 95% power with alpha = 0.05. For the neuroimaging component of this study, we determined baseline sample size from a prior fMRI study of pain in AD as well as preliminary functional connectivity results (Cole et al., 2006, 2011). Initial power calculations yielded necessary *n* = 13 per group (β = 0.95, Cohen’s *d* = 1.49, α = 0.05). However, considering the resting-state nature of this study, and in order to assess connectivity differences within the AD group (i.e., mAD vs. sAD), we reasoned that a sample size to ~20/group with recruitment of semi-equal numbers of mAD and sAD patients to be sufficient.

### Statistical Methods

#### Behavioral Analysis

Generalized linear modeling (GLMM: Generalized linear mixed modeling) in SPSS™ (Version 22.0, Armonk, NY, USA: IBM Corp) determined impact of level-two effects (subject group) on level-one effects (PAINAD score, HR change), with subject and stimulus level as predictors. We analyzed all behavioral results with two subject groupings: first the entire HS and entire AD groups were compared, and second the AD group alone was analyzed, by dividing into mAD and sAD subgroups. This was done to allow examination of disease severity effects separately from disease presence effects. GLMM accounts for repeated measures (trials) and covariates of non-interest (age, gender). PAINAD scores were each recoded into a set of four clustered scores to improve modeling of its non-normal distribution. HR changes were similarly recoded to reflect no change (0), positive response (+1), or negative response (−1) for GLMM testing. Significant “group” (HS vs. AD, or mAD vs. sAD) or “group × stimulus level” interaction effects (*p* < 0.05) were followed-up with *post hoc* nonparametric Mann-Whitney U independent samples testing. Original data (i.e., non-recoded) were utilized for *post hoc* tests between groups by stimulus level; the latter occurred for PAINAD scores and HR responses. As this study was focused on severity dependent effects of AD on HR pain responsiveness we analyzed differences in HR responsiveness between AD subgroups. Finally, we computed effect sizes based on overall group means for the two primary outcomes of interest: for overall mean PAINAD score between HS and AD Glass’ *delta* was computed as standard deviations were not similar; for overall mean HR responses between mAD and sAD Hedges *g* was computed given sample sizes differences between these subgroups.

The aforementioned analyses were followed up with testing of three summary measures between groups. Specifically, each subject’s PAINAD scores and HR responses were examined across stimulus levels to determine slope of change, intercept of the stimulus-response relationship, and mean responsiveness (average measured PAINAD scores and HR changes). These summary measures were explored in a prior study (Kunz et al., 2004) involving multiple pain intensities, though in the context of pain self-report. The measures generally represent indices of degree of reactivity, response threshold, and average amplitude of response, respectively. Again, as we focused on severity dependent effects of AD on HR responses, only AD subgroups were included in HR summary measure analyses. Group-level analyses of summary variables took place via MANOVA testing with age and gender again included as covariates of non-interest. Significant group effects led to subsequent inclusion in rs-fMRI analyses to determine connectivity-behavioral relationships.

#### fMRI Analysis

##### Basic rs-fMRI processing

Individual subject fMRI standard pre-processing was carried out using FMRI Expert Analysis Tool (FEAT) Version 6.00, part of FMRIB’s Software Library (FSL^1^). Steps included: removal of the first two volumes due to enhanced longitudinal magnetization in the first few scans; brain extraction (Smith, 2002); motion correction (Jenkinson et al., 2002); spatial smoothing (FWHM 5 mm); and high pass temporal filtering (sigma 100 s). Individual subject functional scans were nonlinearly registered to MNI space via structural scans (Jenkinson and Smith, 2001; Jenkinson et al., 2002; Andersson et al., 2007a,b). Because meaningful neuronal signal was recently found to be associated with frequencies beyond 0.08 Hz (Niazy et al., 2008; Smith et al., 2008; Cole et al., 2010), low-pass filtering was not employed. Instead, single subject independent components analysis (ICA; Cole et al., 2010; Boubela et al., 2013), using Multivariate Exploratory Linear Decomposition into Independent Components, Version 3.13 (MELODIC; Beckmann and Smith, 2004), was employed to check for and regress out individual time course artifacts. Resulting components for each subject were visually inspected to sort out those likely representing neuronal signal vs. physiologic and/or motion artifact in accordance with Kelly et al. (2010). Artifactual components were then regressed out of each subject functional time series, resulting in individual subject denoised datasets.

Voxelwise regressors were then computed to remove effects of regional atrophy on connectivity measures. For each subject, individual anatomical T1 scans, already in MNI space, were segmented using FSL’s FAST (Zhang et al., 2001) to obtain gray matter (GM) partitions. Individual Jacobian maps were then calculated using warp coefficients produced during nonlinear registration of subject anatomical scans. The GM partitions produced by FAST were then modulated by the Jacobian maps to reflect areas of local expansion or contraction of atrophied regions. Individual Jacobian modulated GM partitions were then merged into a single 4D file to be used as a voxelwise regressor of non-interest for group analyses.

##### General linear model creation

General linear models (GLMs) were then created to test significant associations of resting connectivity with significant behavior summary measures. Each GLM included regressors of interest (i.e., a significant behavioral summary measure) and non-interest (age, gender, voxelwise atrophy measures). Age and gender covariates were demeaned prior to GLM entry. The first set of GLMs were specific to PAINAD summary measures that reached significant differences between HS (*n* = 24) and AD (*n* = 20) groups (Figure 1, bottom blue box). Here a continuous covariate interaction model was designed to assess potential interactions of connectivity and behavioral differences (i.e., interaction effects between group and PAINAD summary measures; PAINAD measures were input as continuous variables). The second set of GLMs probed potential connectivity associations between HR-related summary measures found to be different between mAD and sAD subgroups (Figure 1, bottom red box; HR measures were input as continuous variables). Because our scanned sAD subject sample was limited, a between-groups (i.e., mAD vs. sAD) analysis was not performed. Rather, a within-AD group design was utilized for HR-related GLMs to assess connectivity along a spectrum of AD severity. Once all GLMs were created, further steps were taken prior to group analyses.

##### Group ICA

Next, we created to Group-level ICAs (GICAs) using MELODIC (Beckmann and Smith, 2004). Two GICAs occurred, one for each type of GLM: GICA1 (for PAINAD analyses, Figure 1 middle blue box) included all scanned subjects of both groups; GICA2 included all 20 AD subjects (Figure 1, red boxes). Via MELODIC, principal component analysis (with automatic estimation of dimensional number and variance normalization) allowed for denoised single-subject resting-state fMRI time courses to be temporally concatenated and decomposed into independent spatial maps characteristic of the included study sample (Hyvärinen, 1999; Minka, 2000; Beckmann and Smith, 2004). During this process, non-brain voxels were masked. Estimated component maps were thresholded (*p* > 0.5) by fitting a mixture model to the histogram of intensity values (Beckmann and Smith, 2004). This process resulted in 28 components for GICA1 and 30 for GICA2. Visual comparison to “canonical” RSNs (Smith et al., 2009) indicated which GICA components were neuro-anatomically plausible.

##### Group-level rs-fMRI analyses

FSL’s dual regression technique (Filippini et al., 2009) was then employed to generate subject-specific versions of each GICA spatial map and associated time series. For each subject, all estimated component spatial maps from GICA were regressed (as spatial regressors) into the subject’s 4D space-time dataset, resulting in subject-specific time series, one for each GICA spatial map. Next, individual subject time series were regressed (as temporal regressors) back into a single 4D dataset, resulting in a set of subject-specific spatial maps, again one for each GICA spatial map.

**Figure 2:**
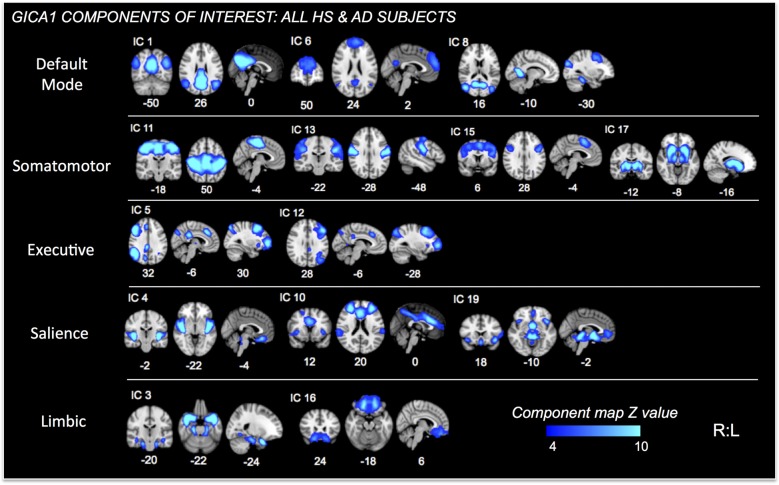
Independent Components (ICs) of interest resulting from Group ICA of all HS and AD subjects. These ICs were used in permutational tests exploring relationships between connectivity and PAINAD score differences between groups. ICs of interest determined via consideration of brain regions/networks typically associated with pain, emotion and behavior. Resultant ICs of non-interest, as well as those representing non-neuronal signal not shown. MNI coordinates (*x*, *y*, *z*) shown below each map.

**Figure 3:**
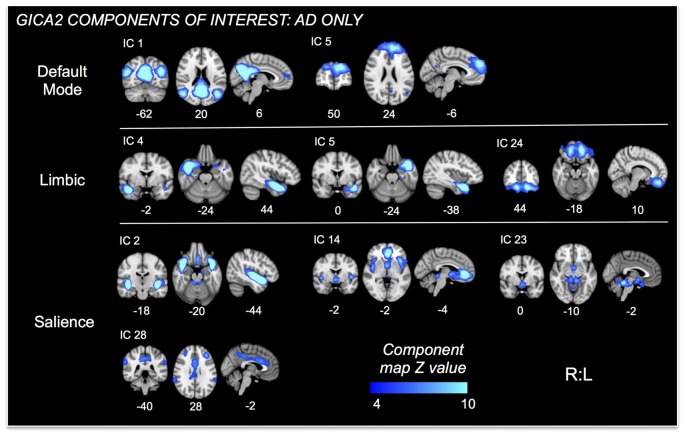
ICs of interest resulting from Group ICA of only AD subjects. These ICs were used in permutational tests exploring relationships between connectivity and HR change differences between mAD and sAD patients. ICs of interest determined via consideration of brain regions/networks typically associated with autonomic responses and regulation. Resultant ICs of non-interest, as well as those representing non-neuronal signal not shown. MNI coordinate shown below each map.

Further analysis was limited to those components whose spatial distributions closely matched RSNs comprised of structures associated with pain processing (Kong et al., 2010, 2013; e.g., salience, default mode, executive/fronto-parietal, somatomotor, and limbic RSNs or related structures—visualized in Figure 2 for GICA1) and the CAN (Beissner et al., 2013; e.g., salience, default mode, temporal limbic—visualized in Figure 3 for GICA2). Each GICA’s selected components were subjected to nonparametric permutation-based testing via FSL’s randomize (Nichols and Holmes, 2002; Winkler et al., 2014) tool (10,000 permutations), with threshold-free cluster enhancement (TFCE; Smith and Nichols, 2009). GLMs were then incorporated to test the relationship between connectivity of selected components and summary behavioral measures. Resultant spatial maps assessed connectivity between the RSN of interest and all voxels of the brain. We addressed the issue of multiple comparisons by controlling family-wise error (FWE) with a threshold of *p* < 0.05.

To examine connectivity strictly between RSNs of interest, we utilized the FSLNets package^2^ as implemented in Matlab^3^. For each RSN of interest individual subject time courses were first extracted and normalized to their standard deviations. Artifactual components and those not of interest were regressed out of each subject’s time course. Next, subject-level network correlation matrices were generated based on the residual time series of each component (L1-regularized, λ = 10). Specifically, partial correlations were utilized, as they are thought to better assess “direct” network connections, in comparison to full correlations (Smith, 2012). Each matrix’s partial r-coefficients were then z-transformed and subjected to autocorrelation correction. Finally, individual subject correlation matrices were averaged to form group level matrices. The group-level matrix was then pre-masked with a *t*-value >8, preventing connections that were not strong on average across all subjects from being tested. Nonparametric permutation testing on networks of interest occurred again via FSL’s randomize (5000 permutations) and GLM integration as outlined above. Multiple comparison control was applied through FWE correction, thresholded at *p* < 0.05.

Because HR connectivity analyses were within-group, we next performed a discriminant classification analysis via SPSS to determine which connectivity result best differentiated AD severity-dependent effects on HR responses. Discriminant analyses allow for prediction of categorical dependent grouping variables through a regression-style analysis of continuous independent predictor variables. The goal is to create a linear discriminant function that best differentiates dependent grouping variables. Beta-values were first extracted from significant clusters and then entered as independents, while AD subgroup designation (i.e., mAD or sAD) was set as the grouping variable. To ensure that data met analysis assumptions of variance homogeneity, Box’s M was calculated. Prior probabilities were then computed from group size as subgroup numbers were not equal. Discriminant scores were cross-validated using a leave-one-out classification approach. Finally, Wilk’s Lambda and canonical correlations were calculated to determine which predictor variable contributed the most in differentiating AD subgroups. In assessing classification results, the best predictor has the highest correlation, the lowest Wilk’s Lambda, and an associated significance level *p* < 0.05.

## Results

### Demographics

Subject demographics are found in Table 1. No differences in age and gender between HS and AD groups were found. Impaired cognition was confirmed in the AD group, as seen by MMSE score comparisons to HS (*F* = 77.0; *p* < 0.001). SIB-S scoring further confirmed significant cognitive decline between mAD and sAD subgroups (*F* = 36.4; *p* < 0.001). AD subjects had greater CSDD scores than HS (*F* = 81.2; *p* < 0.001). However, no AD subjects had CSDD scores indicative of depression. FAQ testing in patients yielded worsening IADL abilities (i.e., increased score) with increasing severity of AD (*F* = 17.5; *p* = 0.001). No differences in baseline HR were found between HS and AD (*F* = 0.36; *p* = 0.56), nor mAD and sAD subgroups (*F* = 0.33; *p* = 0.68). A greater percentage of mAD subjects were on AChEI medication than sAD, but this difference reached only marginal significance (Chi-Sq = 3.5; *p* = 0.06). Finally, AD patients were more likely to be on an SSRI antidepressant than HS (Chi-Sq. = 10.9; *p* = 0.001). There were no differences in SSRI use between mAD and sAD patients (Chi-Sq. = 0.59; *p* = 0.44).

### Behavioral Testing Results

**Figure 4:**
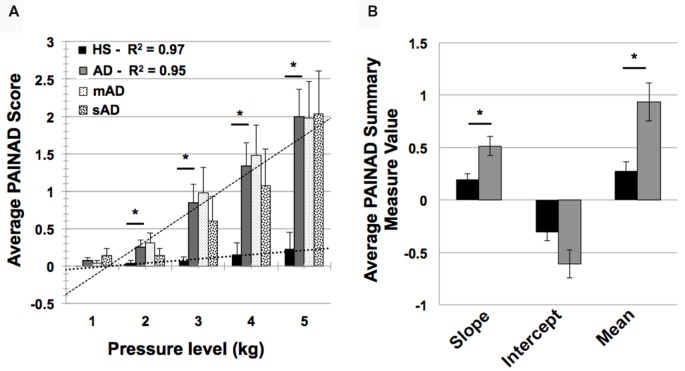
Group level behavioral pain responses (Pain Assessment in Advanced Dementia, PAINAD scale). **(A)** Average PAINAD scores for each pressure intensity (kilograms, kg) among HS and full AD groups; mild/moderate and severe (mAD and sAD) subgroups are also included. Associated linear regression lines are superimposed for HS and AD groups, allowing for visualization of slope of change and intercept across stimulus levels. **(B)** All PAINAD summary measures visualized as group averages, including slope of change, intercept and mean response. Asterisk indicates significant group difference at *p* ≤ 0.05 between HS and AD groups. Error bars represent standard error of the mean (SEM).

In testing HS and AD differences in PAINAD scores, we found a significant main effect of group and a group × stimulus level interaction (*F* = 31.9, 171.2; *p* < 0.001, respectively). As indicated in Figure 4A, *post hoc* Mann-Whitney U testing indicated that AD subjects had greater PAINAD scores for 2–5 kg pressure levels (Standardized Test Statistic, STS = 2.4, 3.6, 2.8, 2.6; *p* = 0.02, <0.001, 0.005, 0.009 respectively). PAINAD responses were not significantly different between mAD and sAD patients (main group effect *F* = 0.03, *p* = 0.86; group × stimulus intensity *F* = 1.8, *p* = 0.08; see Figure 4A). Summary measure testing across stimulus levels for PAINAD scores are visualized in Figure 4B. Here, significant between group effects for slope (*F* = 11.4; *p* = 0.002) and mean PAINAD score (*F* = 9.3; *p* = 0.004) were found. The intercept variable failed to reach significance (*F* = 3.4; *p* = 0.07). Effect size (Gates’ *delta*) for overall mean PAINAD score difference between HS and AD groups was measured at 1.47, indicating a very strong effect.

**Figure 5:**
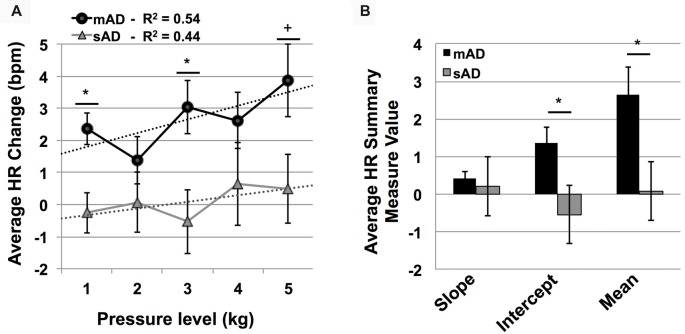
Group level autonomic pain responses (HR in beats per minute, bpm). **(A)** Average HR changes from baseline (bpm) for each pressure intensity (kilograms, kg) among mild Alzheimer’s (mAD; black circles) and severe Alzheimer’s (sAD; gray triangles) patient sub-groups. Associated linear regression lines are superimposed, allowing for visualization of slope of change and intercept across stimulus levels. **(B)** HR response summary measures across pressure levels, including slope of change, intercept and mean response. Asterisk (*) indicates significant group difference at *p* ≤ 0.05. Plus (+) indicates marginal significance (*p* < 0.06). Error bars represent SEM.

Advanced AD patients showed reduced HR responses, compared to more mild patients, with significant group and group × stimulus level interaction effects (*F* = 5.7 and 801.9; *p* = 0.004, <0.001, respectively). As indicated by Figure 5A, *post hoc* Mann-Whitney U testing found significantly lower HR responses in sAD subjects, compared with mAD subjects, for stimulus levels 1 and 3 kg, and marginally so at 5 kg (STS: 2.7, 2.3, 1.9; *p* = 0.005, 0.019, and 0.056, respectively). Summary measure testing across stimulus levels for HR responses are visualized in Figure 5B. Here, significant effects for intercept (*F* = 4.5; *p* = 0.05) and mean (*F* = 6.6; *p* = 0.02) HR responses were found. Slope of HR responses were not different between AD subgroups (*F* = 1.4; *p* = 0.25). Effect size (Hedge’s *g*) for overall mean HR response difference between mAD and sAD sub-groups was measured at 1.03, indicating a very strong effect.

### Functional Connectivity Results

**Figure 6:**
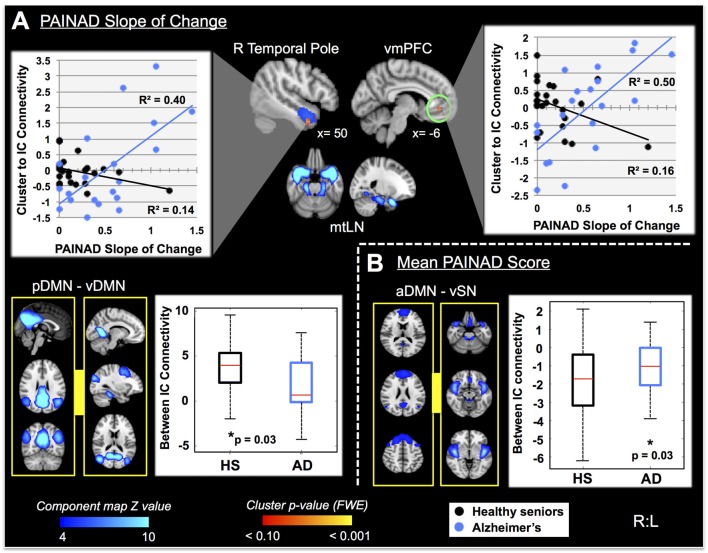
Differential resting state network (RSN) functional connectivity (from GICA1) associated with greater PAINAD slope of change and mean PAINAD scores in AD patients vs. HS. RSNs are presented in blue, while clusters are red-yellow (encircled in green). **(A)** Top, significant interaction effects for group (HS vs. AD) × PAINAD slope of change; top—greater connectivity in AD vs. HS between right (R) temporal pole and ventromedial prefrontal cortex (vmPFC) clusters to a temporal limbic network (TLN, IC3; clusters thresholded at *p* < 0.10 for visualization purposes); the interaction is visualized on the right with connectivity (beta values) plotted against PAINAD slope with superimposed linear regression lines; bottom—reduced connectivity in AD vs. HS between posterior and ventral components of the DMN (pDMN and vDMN, ICs 1 and 8, left); group connectivity strength differences (Z-transformed partial correlations) visualized on the right. **(B)** Right—significant interaction effect for group × mean PAINAD score with greater connectivity in AD vs. HS for an anterior DMN (aDMN, IC 6) component and ventral SN (vSN, IC 4) component; left—group connectivity strength differences visualized (Z-transformed partial correlations). Coordinates are MNI space.

Fourteen RSNs from GICA1 (visualized in Figure 2) deemed pain-relevant (Kong et al., 2010, 2013) were entered into dual-regression/randomize and FSLNets analyses to examine how group differences in slope of change and mean PAINAD scores across stimulus levels were related to group differences in RSN connectivity. A significant interaction effect was found for group × PAINAD slope of change within the TLN (GICA1—IC 3), for a right temporal pole cluster (*p* < 0.05, FWE; Table 2, visualized in Figure 6A, top-left). As indicated by Figure 6A, connectivity between the TLN and right temporal pole cluster was greater in more behaviorally reactive AD patients, with the opposite occurring in HS. The same effect occurred between the TLN and a cluster in the vmPFC (*p* < 0.05, FWE corrected; Table 2; visualized in Figure 6A, top-right). Using FSLNets we also found a group × PAINAD slope interaction associated with connectivity between posterior DMN (pDMN, GICA1—IC1) and ventral DMN (vDMN, GICA1—IC8) components. Here, greater PAINAD slope of change in AD patients was associated with reduced pDMN-vDMN connectivity, compared to HS (*p* = 0.03, FWE; Table 2, visualized in Figure 6A, bottom-left). Figure 6B shows connectivity results pertaining to mean PAINAD scores. Here, greater average pain behavioral scores in AD patients were associated with increased connectivity strength between anterior DMN (aDMN) and ventral salience network (vSN; GICA1—ICs 6 and 4, respectively) in AD, compared to HS subjects (*p* = 0.03, FWE; Table 2). Further scrutiny of this result indicated greater PAINAD scores in AD patients were associated with reduced anti-correlation between the aDMN and vSN.

**Table 2:** Connectivity between cluster-resting state networks or between networks significantly correlated with Pain Assessment in Advanced Dementia (PAINAD) score differences between healthy seniors (HS) and Alzheimer’s disease (AD) and heart rate (HR) changes within AD subjects.

IC #	RSN	Statistical connectivity contrast	P_FWE_	#Vox	MNI coord.			Hemi	Cluster location
					*X*	*Y*	*Z*		
**PAINAD slope**
IC 2	MesTemp	Interaction HS < AD	0.04	18	50	2	−32	R	Temporal pole
		Interaction HS < AD	0.04	12	−6	42	−8	L	vmPFC
ICs 1 and 8	pDMN —vDMN	Interaction HS > AD	0.03	-	-	-	-	-	Between ICs
**Mean PAINAD**
ICs 4 and 6	aDMN —vSN	Interaction HS < AD	0.03	-	-	-	-	-	Between ICs
**HR change intercept**
IC 1	pDMN	Pos HR	0.002	270	2	−66	36	R	Precuneus
**Mean HR change**
IC 1	pDMN	Pos HR	0.005	166	2	−66	32	R	Precuneus
IC14	vSN2	Neg HR	0.021	58	−2	42	−16	L	vACC/vmPFC

*IC, independent component; RSN, resting state network; P_FWE_, p-value of cluster after family-wise error correction; #Vox, number of voxels within the cluster; MNI coordinate, cluster maximum in mm; Hemi, hemisphere (L, Left vs. R, Right); MesTemp, Mesial Temporal component; pDMN, posterior default mode network component; vSN2, #2 ventral salience network component; Interaction, continuous covariate interaction effect between groups; Neg/Pos HR, negative or positive statistical heart rate correlation with connectivity; vmPFC, ventromedial prefrontal cortex*.

**Figure 7:**
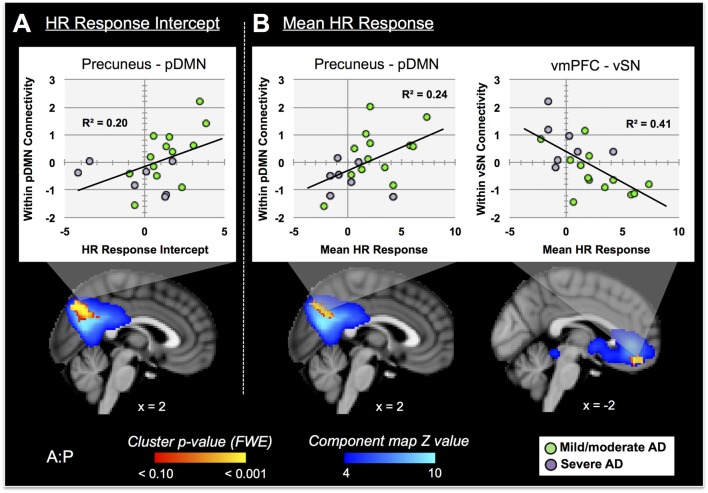
RSN connectivity significantly correlated with HR response intercept and mean HR response within AD subjects. Associated ICs are presented in blue, clusters are red-yellow. Mean cluster beta-values, representing cluster–network connectivity strength for each subject, are plotted against HR responses (mild/moderate AD (mAD) green circles, sAD purple circles). **(A)** Top—plot of positive association between precuneus—posterior DMN component (pDMN, IC 1) and HR change intercept; bottom—visualization of the cluster. **(B)** Top—plots of positive and negative associations of precuneus–pDMN and vmPFC–ventral SN (vSN, IC 14) component, respectively, for mean HR change; bottom—visualization of clusters. Clusters visualized at *p* < 0.05, family-wise error (FWE). Coordinates are MNI space.

Nine autonomic-related RSNs from GICA2 (Figure 3) were selected for HR-related analyses of intercept and mean response differences between mAD and sAD patients. With respect to intercept, connectivity within a pDMN network (GICA2—IC1), specifically a cluster in the precuneus, positively correlated with HR responses of AD subjects (*p* = 0.002, FWE; Table 2; visualized in Figure 7A). Similarly, within pDMN connectivity (precuneus cluster; *p* = 0.005; Table 2; visualized in Figure 7B, left) positively correlated with slope of change of AD subjects. In contrast, connectivity within the vSN (GICA2—IC14) to a left vmPFC cluster (*p* = 0.021, FWE; Figure 7B, right; Table 2) was negatively correlated with AD subject mean HR changes. No significant between-network connectivity relationships were found for HR changes via FSLNets.

In performing discriminant classification analyses of HR connectivity clusters we found that homogeneity assumptions were met (Box’s M: *F* = 5.3, *p* = 0.66). The results of discriminant classification analyses are found in Table 3. Examination of canonical correlations and Wilk’s Lambda results indicated that the negative correlation of connectivity of the vSN to the vmPFC by far best discriminated between AD severities.

**Table 3:** Significant correlations between resting state network connectivity and pain responses.

	Summary of canonical discriminant functions		
	Canonical correlation	Wilks’ lambda	*p*
**HR change intercept**
pDMN —precuneus	0.43	0.81	0.057
**Mean HR change**
pDMN —precuneus	0.45	0.80	0.045*
vSN —vmPFC	0.58	0.67	0.007*

pDMN, posterior default mode network component; vSN, ventral salience network component; vmPFC, ventromedial prefrontal cortex. Bold* considered significant at *p* < 0.05.

## Discussion

### Pain Behavioral Findings

#### Pain Behaviors in AD

Several studies have found greater degrees of acute pain behaviors in AD patients, relative to HS (Cole et al., 2006; Kunz et al., 2007; Jensen-Dahm et al., 2014; Beach et al., 2015, 2016). We confirmed this in our sample of patients for all but the lowest pressure level. When examining summary parameters of pain behaviors (i.e., PAINAD scores), we found that patients showed both a greater stimulus-response slope and greater mean PAINAD scores, compared to controls. Thus, evidence for greater pain reactivity in patients may be found across single and multiple stimulus levels. Increased pain behaviors in patients compared to controls were associated with a number of connectivity differences.

#### rs-fMRI Correlates of Increased Pain Behaviors in AD

First, connectivity both within the TLN and between the TLN and a cluster in the vmPFC correlated positively with pain behavior reactivity (PAINAD score slope of change) in AD subjects, but negatively in HS. The TLN elaborated by GICA1 is comprised of bilateral hippocampal gyrus, amygdala and temporal pole, which are among the earliest structures to be affected by AD pathology (Braak and Braak, 1995; Braak et al., 1999). These TLN structures are implicated in pain-related episodic memory, negative affect and associated behaviors, and socio-emotional regulation, respectively (Quirk and Gehlert, 2003; Olson et al., 2007). Likewise affected by AD (Chu et al., 1997; Tekin et al., 2001), the vmPFC is involved in implicit contextual appraisal of affective stimuli, based on long-term memory (Roy et al., 2012). The vmPFC utilizes this appraisal mechanism to regulate both pain and general affect through inhibition of the amgydala via dense medial temporal projections (Quirk and Gehlert, 2003; Ghashghaei et al., 2007; Bingel et al., 2008; Kunz et al., 2011; Shackman et al., 2011; Lee et al., 2012; Etkin et al., 2015; Motzkin et al., 2015). In healthy individuals amygdala activity is associated with increased facial expression during negative affect while vmPFC activity is associated with the opposite (Lanteaume et al., 2007; Kunz et al., 2011; Heller et al., 2014). However, in AD the relationship between these structures changes, potentially as a part of a general limbic and prefrontal mediated mechanism of functional reallocation to compensate for impaired cognition, including memory-dependent appraisal processes (Grady et al., 2001; Benedetti et al., 2006; Rosenbaum et al., 2010). This may partly explain why there is increased connectivity between the vmPFC and TLN in behaviorally over-responsive AD patients. In one study, damage to the vmPFC was associated with greater connectivity of the amygdala to temporal pole (Motzkin et al., 2015), consistent with increased pain behavioral reactivity in AD patients. This suggests AD-mediated vmPFC dysfunction may enhance TLN (likely amygdala) mediated aversion processing and associated behavioral output. These findings may extend to affective displays beyond pain; studies have found increased negative emotional responses to threat, particularly with respect to facial expression, in AD patients (Smith, 1995; Burton and Kaszniak, 2006; Henry et al., 2009a,b; Seidl et al., 2012). Important to this discussion, greater emotional reactivity by AD patients is associated with atrophy or dysfunction in affect-associated structures (Starkstein et al., 1995; Lyketsos et al., 2002; Bruen et al., 2008; Sturm et al., 2013). For example, greater neurofibrillary tangle burden in the vmPFC is associated with increased agitation and aberrant motor behaviors in AD patients (Tekin et al., 2001).

Second, greater PAINAD slope of change in patients was associated with reduced connectivity between posterior and ventral DMN subcomponents. Subdivisions of the DMN are each associated with distinct cognitive functions (Uddin et al., 2009; Andrews-Hanna et al., 2010; Kim, 2012): aDMN, comprised primarily of dorsomedial PFC (dmPFC), with self-referential processing; pDMN, primarily posterior cingulate/precuneus and lateral parietal cortex, with internally directed thought and autobiographical memory; and the vDMN, containing retrosplenial, parahippocampal and various associative cortices, with episodic-memory based future decision making. In turn, the connectivity of each DMN subdivision is affected by AD (Damoiseaux et al., 2012). Reduced connectivity between pDMN and vDMN in AD patients could contribute to greater pain reactivity (i.e., PAINAD slope of change) by impairing pain-related working memory during internally directed attention; appropriate contextual appraisal of acute pain by the vmPFC could thus be undermined.

Next, greater mean PAINAD scores in patients, compared to controls, were associated with reduced anti-correlation between aDMN and vSN. In contrast to the DMN, the SN is implicated in emotional reactivity, and is described as directing attention to important internal or external events, e.g., pain (Critchley, 2005; Seeley et al., 2007; Hänsel and von Känel, 2008; Rolls and Grabenhorst, 2008; Etkin et al., 2015). Often the SN and DMN are segregated into opposing “task positive” and “task negative” networks, which subserve recurrent switching between externally and internally directed attention, respectively (Fox et al., 2005; Fransson, 2005). Even in resting conditions the DMN and SN are typically anti-correlated (Fox et al., 2005; Seeley et al., 2007). However, in the context of pain their relationship becomes more complex. For example, the DMN remains active during periods of high pain, compared to low pain or tactile somatosensation (Mantini et al., 2009; Kong et al., 2010; Ter Minassian et al., 2013); this has been proposed to represent internal mentation directed toward threatening (painful) stimuli (Kong et al., 2010; Ter Minassian et al., 2013). In AD the relationship between the DMN and other RSNs becomes increasingly dysfunctional. Especially pertinent to our results are prior findings of reduced resting anti-correlation between the DMN and SN as well as greater DMN-related activity during task conditions in AD patients, compared to controls (Lustig et al., 2003; Cole et al., 2006; Wang et al., 2007; Brier et al., 2012). Interestingly, reduced functional segregation between the DMN and SN is hypothesized to partly account for the attentional deficits and distractibility seen early on in AD (Perry and Hodges, 1999; Perry et al., 2000). Thus, our aDMN-vSN related results suggest a propensity of AD patients, compared with HS, to attend to and inwardly evaluate potentially noxious stimuli more than HS. Further, the dmPFC (the primary anatomical site of the aDMN), is implicated in automatic and voluntary affect regulation (Padberg et al., 2001; Lee et al., 2012; Ray and Zald, 2012; Buhle et al., 2014) and inhibition of pain-related facial expression (Kunz et al., 2011; Karmann et al., 2016). Increased pain behaviors in AD patients may thus also be related to dysfunctional prefrontal affective and salience mechanisms (Kunz et al., 2007, 2009; Beach et al., 2015, 2016).

Taken together our results suggest that increased pain behavior responsiveness in AD patients involves dysfunctional prefrontal and temporal limbic affective-behavioral regulation, reduced memory-based contextual appraisal, and increased pain-related internal mentation. This putative mechanism is supported by studies by Cole et al. (2006, 2011), who found that AD patients, compared to HS, exhibited greater pain-induced activity and functional connectivity, particularly with respect to the dorsolateral PFC (dlPFC). The dlPFC is part of both the SN and frontal executive networks and engages in explicit, or cognitive based, affective regulation and goal-based decision making (Lorenz et al., 2003; Ridderinkhof et al., 2004; Etkin et al., 2015). The dlPFC and vmPFC are both functionally and anatomically connected, such that the former modulates the activity of the latter (Hare et al., 2009). Because of poor executive function and altered executive RSN connectivity in AD, particularly with respect to the dlPFC (Seeley et al., 2007; Kaufman et al., 2010; Agosta et al., 2012), emotional display, as governed by the vmPFC, may increase via impaired dlPFC-mediated inhibitory control (Hare et al., 2009; Mograbi et al., 2012). Here we found no results directly tied to dlPFC connectivity. This may pertain to our use of rs-fMRI, rather than an activation-based paradigm such as that used by Cole et al. (2011) which could induce pain evaluative processing by this region. Future work could involve investigating seed-based connectivity measures between prefrontal cortical regions and their associations with pain-related measures (e.g., behavior, pain report). In sum, altered resting connectivity of vmPFC to limbic regions and functional desegregation of prefrontal default mode to salience/medial pain networks may preclude normal regulation of pain behaviors in AD patients posited to occur during active pain processing (Kunz et al., 2011, 2015; Karmann et al., 2016). Impaired pain behavior inhibitory mechanisms may also contribute to prior findings of AD patients showing increased subjective pain ratings and reduced pain tolerance (Cole et al., 2006; Oosterman et al., 2009; Jensen-Dahm et al., 2014; Beach et al., 2015; Karmann et al., 2016).

### Autonomic Pain Response Findings

#### HR Responsiveness to Pain in AD

Altered autonomic pain responses are also commonly seen in AD patients, particularly with disease progression (Porter et al., 1996; Rainero et al., 2000; Kunz et al., 2009; Beach et al., 2015). In our scanned sample we confirmed reduced HR responses in sAD patients (MMSE <11). Summary measures showed that reduced autonomic responses included both threshold and mean HR response across pressure levels; thus, a higher stimulus threshold is required to obtain even a modest increase in HR as AD progresses (Rainero et al., 2000). However, altered autonomic responsiveness in AD is likely not specific to pain; a number of studies have found evidence of general autonomic dysfunction in patients that worsens with advancing disease and involves impaired sympathetic responsiveness, e.g for valsalva maneuver, isometric handgrip, sympathetic skin response, and deep breath 30:15 ratio (Algotsson et al., 1995; Zulli et al., 2005; Zakrzewska-Pniewska et al., 2012; Jensen-Dahm et al., 2015; Nonogaki et al., 2017). Autonomic dysfunction in AD likely stems from its early and progressive effects on autonomic related cortex and subcortical nuclei (Chu et al., 1997; Rüb et al., 2001). However, few studies have investigated the functional neural correlates of altered autonomic function in patients particularly with respect to pain.

#### rs-fMRI Correlates of Reduced Pain-Related HR Responses in AD

Turning to our imaging results, we first found that a precuneus cluster within the pDMN was positively correlated with HR response intercept in AD subjects. A nearly identical result occurred with respect to mean HR response. Because of its putative role in passive internal and environmental monitoring (Buckner et al., 2008; Deco et al., 2011; Babo-Rebelo et al., 2016) it is logical that DMN function is associated with parasympathetic predominance (Nagai et al., 2004a; Wong et al., 2007; Napadow et al., 2008; Fan et al., 2012). Cortical arousal, as indexed by alpha EEG power, is also associated with DMN activity at rest (Knyazev et al., 2011; Mayhew et al., 2013a,b). Together, these processes allow for a calm, but alert brain that can perform necessary internal monitoring and respond appropriately to physiologic demands. In AD patients, delta and theta power, associated with sympathetic suppression (Baharav et al., 1995; Brandenberger et al., 2001; Yang et al., 2002; Kuo and Yang, 2004), increasingly predominate during wakefulness as the disease progresses (Petit et al., 2004; Tsolaki et al., 2014). There is also a well-known degradation of DMN function as AD advances (Greicius et al., 2004; Damoiseaux et al., 2012). Interestingly, Benedetti et al. (2004) found the degree of resting delta power increased with AD severity; delta power was negatively correlated with autonomic pain responses in patients and controls. Overall, these findings suggest that impaired resting cortical arousal, in association with increasingly reduced DMN function, is in part associated with reduced autonomic pain responses in AD patients. Our finding of a positive association of within-pDMN connectivity to both HR response intercept (threshold) and mean HR response (magnitude) in AD patients supports this notion.

Next, we found that network connectivity within the vSN, specifically a cluster in the left vmPFC, was negatively correlated with mean HR responses of patients. Discriminant classification analysis of these connectivity results determined that the vmPFC cluster relationship with mean HR change best differentiated patients based on disease severity (i.e., mAD vs. sAD), compared to within-pDMN connectivity. This vmPFC finding is particularly compelling. The vmPFC, part of the CAN (Critchley, 2005; Beissner et al., 2013), is associated with modulating autonomic balance to enhance vagal output (Hilz et al., 2006; Ziegler et al., 2009; Motzkin et al., 2014). The vmPFC is affected early and progressively by AD (Chu et al., 1997; Van Hoesen et al., 2000; Rüb et al., 2001) and was hypothesized by Chu et al. (1997) to act as a key contributor to their autonomic disturbances. Lesion studies also provide evidence of hemispheric specialization of this region, whereby right vmPFC inhibits sympathetic drive and left vmPFC activates parasympathetic output (Damasio et al., 1990; Bechara et al., 1999; Hilz et al., 2006). Progressive left-sided vmPFC dysfunction as AD advances may thus predispose patients toward a parasympathetic predominant autonomic pain response. Discriminant classification results here suggest these functional changes may be the primary driver of altered autonomic pain responses in AD.

### Pain Behavior-Autonomic Disconnect

Interactions between nociceptive and autonomic systems are widespread, allowing for cohesive cognitive, affective, autonomic and somatomotor responses to internal or external environmental demands (Critchley, 2005; Benarroch, 2006). Theories on the influence of internal bodily states on brain and behavior describe how the vmPFC, along with several other structures like the insula, hypothalamus, amygdala, PAG and various brainstem nuclei perform interoceptive functions (i.e., monitor the body’s internal physiological condition) to bias perception, emotion and behavior (Critchley et al., 2004; Critchley and Harrison, 2013; Damasio and Carvalho, 2013). We found no differences in behavioral responsiveness to pain stimuli between mAD and sAD patients here or in prior studies (Beach et al., 2015, 2016). Thus, the tendency for AD patients, particularly those who are more advanced, to show blunted autonomic pain responses, is a confounding phenomenon. Resolving this disconnect would be advantageous to understanding pain and co-morbid conditions in AD. For example, recent studies suggest both pain and autonomic dysfunction contribute to neuropsychiatric and behavioral symptoms in AD patients (Idiaquez et al., 2002; Ballard et al., 2011). It seems plausible that the pain behavior-autonomic disconnect in AD reflects dysfunction at the neural intersection of affect and its expression, salience detection, internal mentation, interoception and autonomic regulation—i.e., the vmPFC. The vmPFC’s overlapping role in this regard stems from its dense connections to subcortical structures involved in affect generation (e.g., the amygdala; Ghashghaei et al., 2007), interoception and physiologic regulation (e.g., insula, hypothalamus, brainstem; Van Eden and Buijs, 2000), and cognitive control (e.g., dlPFC, dorsal anterior cingulate; Öngür and Price, 2000; Hare et al., 2009). As such, it is implicated in a number of functional networks, including default mode, limbic and salience (Raichle et al., 2001; Nagai et al., 2004b; Seeley et al., 2007; Yeo et al., 2011). Dysfunction of the vmPFC may thus lead to a host of affective, physiologic, and cognitive effects. This is exemplified by patients with direct vmPFC lesions, who show reduced autonomic responses during emotional stimuli, but not neutral stimuli or during resting conditions (Hilz et al., 2006; Motzkin et al., 2014).

We found that the vmPFC had increased connectivity to the TLN in patients with increased pain behaviors. Further, vSN to vmPFC connectivity in association with mean HR pain response was best able to differentiate AD severity. In agreement with a prior hypothesis by Chu et al. (1997), we propose that dysfunction in the vmPFC is a likely a primary contributor to the pain behavior-autonomic disconnect seen as AD advances. Specifically, through mechanisms described above, low to moderate levels of noxious stimulation may confer greater degrees of affective-expressive and attentional processing in patients (Cole et al., 2006, 2011); however this processing may not be able to override the high threshold for a sympathetic response imposed by a dysfunctional vmPFC as AD progresses (Rainero et al., 2000; Beach et al., 2015).

### Limitations and Future Directions

A major strength of this study is its inclusion of AD patients whose impairment ranged from mild to very severe. Also our utilization of multiple resting-state runs provides for greater assurance of reliable connectivity results in our AD group. Nevertheless, the included sample of sAD patients was limited by difficulties inherent to scanning patients with advanced cognitive impairment. Further, while pre-stimulus or RSN connectivity may influence pain or autonomic-related activity, we can only make indirect inferences herein as acute pain responses were not obtained during scanning; the latter may have been further influenced by time between behavioral testing and scanning timing, which was up to several days. Future studies would thus benefit from combining acute pain and resting-state scanning paradigms to examine the pain-related “rest-stimulus” interaction (Northoff et al., 2010) in AD. Because no participants met criteria for current depression CSDD scores were not included as a covariate of non-interest. Nevertheless, as AD patients scored higher on the CSDD, some aspects of depression cannot be completely ruled out as a potential confounder. With respect to autonomic findings, it is possible that use of alternative measures, such as sympathetic skin response or HR variability, may have yielded different results with respect to pain responsiveness and connectivity. Future studies would benefit from examination of neuroimaging correlates of multiple autonomic modalities within and outside of the context of pain in AD. Finally, both permutation-based testing and FWE correction methods were used to reduce risk of Type 1 error at a voxel-wise and RSN level. Nevertheless some caution is necessary in interpreting our results given the number of RSNs (14 from GICA1, 9 from GICA2) whose connectivity was each correlated with two sets of behavioral summary measures (PAINAD slope and mean; HR intercept and mean).

Further experiments would greatly aid in our understanding of neural mechanisms underpinning altered pain in AD. For example: assessing the relationship between regional atrophy and white matter integrity with behavioral and autonomic differences in AD patients and controls; examining pain-related rest-stimulus interactions as described above; a conjunctional analysis of multiple types of behavioral (e.g., in-scanner facial expressions) and autonomic pain responses (HR, sympathetic skin response, HR variability) in patients and controls across multiple pain modalities; and finally, analysis of connectivity associations with subjective pain ratings in patients and controls.

## Conclusion

Examining our results as a whole we find: first, that greater pain behavioral reactivity in AD patients, compared to controls, is associated with altered connectivity in networks and structures associated with affect and the regulation of affective behavioral expression, memory, salience, and internal mentation; second, an increased threshold for and generally reduced sympathetic autonomic response in advancing AD is associated with increasingly dysfunctional connectivity within networks associated with internal mentation/cortical arousal and autonomic regulation; thirdly, the pain behavior-autonomic disconnect seen as AD advances may be rooted in progressive dysfunction of the vmPFC. These findings represent an additional step in understanding the neural mechanisms underlying altered pain responses in AD. They also underscore the necessity for appropriate assessment and treatment of pain in patients with AD, regardless of severity.

## Author Contributions

All authors contributed appropriately with respect to data interpretation, intellectual content and have read/approved the final version of the manuscript. Other specific contributions included the following: PAB was the primary study designer, recruiter, data analyzer and manuscript writer; JTH assisted in data collection and behavioral coding; DCZ provided guidance with respect to fMRI experimental design/analysis and manuscript editing; ACB supervised study design, implementation, analysis and manuscript editing.

## Conflict of Interest Statement

The authors declare that the research was conducted in the absence of any commercial or financial relationships that could be construed as a potential conflict of interest.

## Footnotes

^1^www.fmrib.ox.ac.uk/fsl

^2^http://fsl.fmrib.ox.ac.uk/fsl/fslwiki/FSLNets

^3^http://www.mathworks.com/products/matlab/

## Supplementary Material

FIGURE S1 Force Dial FDK 20 Force Gauge (Wagner Instruments, Greenwich, CT, USA) utilized during behavioral pressure pain testing.
