# Supplementary figures and images for: Altered Behavioral and Autonomic Pain Responses in Alzheimer’s Disease Are Associated with Dysfunctional Affective, Self-Reflective and Salience Network Resting-State Connectivity
Source: Front Aging Neurosci. 2017 Sep 14;9:297. doi: 10.3389/fnagi.2017.00297 (PMC5603705; doi:10.3389/fnagi.2017.00297)

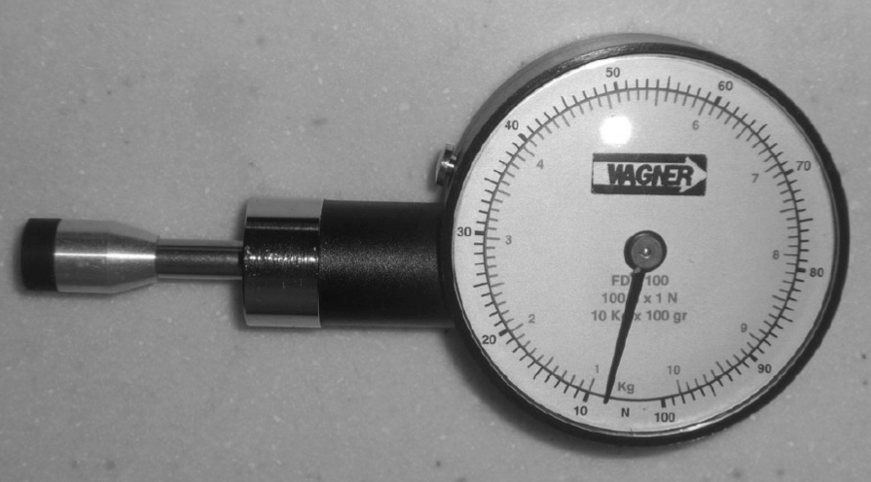

Supplement: FIGURE S1 — Force Dial FDK 20 Force Gauge (Wagner Instruments, Greenwich, CT, USA) utilized during behavioral pressure pain testing. [file Image_1.jpeg]
